# Effect of titanium implants along with silver ions and tetracycline on type I interferon-beta expression during implant-related infections in co-culture and mouse model

**DOI:** 10.3389/fbioe.2023.1227148

**Published:** 2023-10-19

**Authors:** Muhammad Imran Rahim, Syed Fakhar-ul-Hassnain Waqas, Stefan Lienenklaus, Elmar Willbold, Michael Eisenburger, Meike Stiesch

**Affiliations:** ^1^ Department of Prosthetic Dentistry and Biomedical Materials Science, Lower Saxony Centre for Biomedical Engineering, Implant Research and Development (NIFE), Hannover Medical School, Hannover, Germany; ^2^ Biomarkers for Infectious Diseases, TWINCORE, Centre for Experimental and Clinical Infection Research, Hannover, Germany; ^3^ Institute of Laboratory Animal Science, Hannover Medical School, Hannover, Germany; ^4^ Department of Orthopedic Surgery, Lower Saxony Centre for Biomedical Engineering, Implant Research and Development (NIFE), Hannover Medical School, Hannover, Germany

**Keywords:** silver, type I interferon, co-culture, animal model, biomaterial-associated infections, *in vivo* bioluminescent imaging

## Abstract

Type I interferon-beta (IFN-β) is a crucial component of innate and adaptive immune systems inside the host. The formation of bacterial biofilms on medical implants can lead to inflammatory diseases and implant failure. Biofilms elicit IFN-β production inside the host that, in turn, restrict bacterial growth. Biofilms pose strong antibiotic resistance, whereas surface modification of medical implants with antibacterial agents may demonstrate strong antimicrobial effects. Most of the previous investigations were focused on determining the antibacterial activities of implant surfaces modified with antibacterial agents. The present study, for the first time, measured antibacterial activities and IFN-β expression of titanium surfaces along with silver or tetracycline inside co-culture and mouse models. A periodontal pathogen: *Aggregatibacter actinomycetemcomitans* reported to induce strong inflammation, was used for infection. Silver and tetracycline were added to the titanium surface using the heat evaporation method. Macrophages showed reduced compatibility on titanium surfaces with silver, and IFN-β expression inside cultured cells significantly decreased. Macrophages showed compatibility on implant surfaces with tetracycline, but IFN-β production significantly decreased inside seeded cells. The decrease in IFN-β production inside macrophages cultured on implant surfaces with silver and tetracycline was not related to the downregulation of *Ifn-β* gene. Bacterial infection significantly upregulated mRNA expression levels of *Isg15, Mx1, Mx2, Irf-3, Irf-7, Tlr-2, Tnf-α, Cxcl-1,* and *Il-6* genes. Notably, mRNA expression levels of *Mx1, Irf7, Tlr2, Tnf-α, Cxcl1,* and *Il-6* genes inside macrophages significantly downregulated on implant surfaces with silver or tetracycline. Titanium with tetracycline showed higher antibacterial activities than silver. The *in vivo* evaluation of IFN-β expression around implants was measured inside transgenic mice constitutive for IFN-β expression. Of note, the non-invasive *in vivo* imaging revealed a significant decrease in IFN-β expression around subcutaneous implants with silver compared to titanium and titanium with tetracycline in sterile or infected situations. The histology of peri-implant tissue interfaces around infected implants with silver showed a thick interface with a significantly higher accumulation of inflammatory cells. Titanium implants with silver and tetracycline remained antibacterial in mice. Findings from this study unequivocally indicate that implant surfaces with silver decrease IFN-β expression, a crucial component of host immunity.

## 1 Introduction

The application of biomedical implants, such as dental implants, vascular stents, and artificial hips, is increasing every year to improve the lives of millions of people globally (Huebsch and Mooney, 2009). Biomaterials are composed of non-shedding surfaces that make it easier for bacteria to cling onto implants and form pathogenic biofilms (Busscher et al., 2012). Bacteria encased within biofilms produce extracellular polymeric substances (EPS), which provide a dormant lifestyle to bacteria, reducing their susceptibility to antibiotics and the host immune system (Parvizi et al., 2010; Pinto et al., 2020). In addition, biofilms release protein molecules such as hemolysins, leukocidins, nucleases, and endotoxins that interfere with innate and adaptive immune cells (Belibasakis et al., 2015; Ricciardi et al., 2018). The persistence of pathogenic biofilms may cause irreversible tissue destruction in approximately 50% of dental implants, with massive health and economic losses (Grischke et al., 2016). The innate immune system is the first line of defense that recognizes pathogens and is situated to support the initiation of the adaptive immune system. The recognition of pathogens is mediated by receptors (pattern-recognition receptors, PRRs) that alert the immune system and initiate immune responses (Mogensen, 2009). Recent studies have shown bacterial biofilms elicit type I interferon immune response and activate interferon beta (IFN-β) production (Valle et al., 2013a). Type I interferons in healthy situations are essential inducers of complex signaling pathways that manifest antimicrobial host immune responses (McNab et al., 2015). A basal level of type I interferon expression is needed to maintain immune homeostasis (Schaupp et al., 2020; Ayala et al., 2021). Initially, IFN-β were described as products secreted mainly by virus-infected cells. It has now been reported that bacteria induce IFN-β production mostly following the recognition of bacterial nucleic acids or the cell wall component lipopolysaccharide (LPS) by host cells (Boxx and Cheng, 2016). Type I IFN production protects the host from extracellular bacteria (Xiao et al., 2009; Kaplan et al., 2012). Mechanisms for interferon induction against bacteria have been studied mostly by focusing on IFN-β expression. It has been reported that bacterial DNA and RNA mediate the induction of IFN-β through the cytosolic DNA sensor cyclic GMP-AMP synthase (Oldenburg et al., 2012; Andrade et al., 2016). Focusing on IFN-β could be a promising strategy for investigating the host-biofilm interactions. Due to the presence of EPS and reduced metabolic activities, biofilms pose strong resistance to the antibiotics applied systemically. Surface modification of biomaterials by applying antibacterial agents remains a promising strategy to prevent biofilms, as antibacterial agents present directly on implant surface provide localized protection at the peri-implant tissue interface without systemic side effects (Xie et al., 2022). Antibiotics are frequently applied on the implant surface; however, due to the emergence of multidrug-resistant strains, the application of silver (Ag)-based antimicrobials has gained significant interest to control infections from planktonic bacteria and biofilms (Morones-Ramirez et al., 2013; Wang et al., 2020; Wang et al., 2021). Silver ions generate hydroxyl radicals which interfere with bacterial metabolism resulting in the better elimination of the bacteria (Yamanaka et al., 2005; Park et al., 2009). The application of silver on dental implants showed significant antibacterial activity against multispecies biofilms (Noronha et al., 2017; Denis et al., 2022). Host immune cells recognize silver as foreign molecules, and exposure of the silver to these cells could either stimulate or suppress the expression of inflammatory cytokines (Ninan et al., 2020). In this scenario, determining the effect of silver from the surface of titanium implants on IFN-β expression inside the host could provide useful information to understand the effect of these antibacterial agents on inflammatory cytokines. In the present study, silver nitrate and tetracycline were added on the surfaces of titanium implants using the heat mediation method, and then their antibacterial activities and effect on IFN-β production were measured in murine macrophages and a mouse model under sterile and in the presence of bacterial biofilms by a periodontal pathogen, *A. actinomycetemcomitans.* This bacterium was used as it is responsible for biofilm formation around dental implants and associated inflammatory diseases. Moreover, infection of subcutaneous implants with *A. actinomycetemcomitans* induced strong IFN-β expression in transgenic mice (Rahim et al., 2020b). IFN-β expression in transgenic mice was measured using a non-invasive *in vivo* imaging system that facilitated bioluminescent imaging of interferon within the same alive animal at multiple time points.

## 2 Materials and methods

### 2.1 Application of silver and tetracycline on the surface of titanium implants

For *in vitro* evaluations, titanium in the morphology of discs (12 mm diameter and 2 mm thickness, L. Klein SA Switzerland) were used. For *in vivo* investigations, titanium implants in cylindrical porous morphologies (4.5 mm diameter, 7 mm length with 24 pores, each pore 0.5 mm) were used (Rahim et al., 2020b). Silver nitrate (AgNO_3_) or tetracycline suspended in ultrapure water at concentration 1.5 μg/disc were deposited on titanium discs by using heat evaporation at 37°C. The addition of silver or tetracycline on the cylindrical porous titanium implants was done by incubating these implants inside silver nitrate (AgNO_3_; each implant with 250 µL of 500 μg/mL) or tetracycline (250 μL of 500 μg/mL) solutions. Implants dipped inside solutions with silver or tetracycline were incubated at 37°C for 3 days. Implants carrying silver or tetracycline were imaged with a Zeiss Merlin field emission scanning electron microscope (SEM) (EVO MA10), and elemental composition was measured with energy dispersive spectroscopy (EDS) with an element detector from EDAX (Mahwah, New Jersey, United States) (Rahim et al., 2017b). The concentration of silver and tetracycline deposited on cylindrical titanium implants was determined by measuring the light absorption at a wavelength of 225 nm with a Nanodrop ND-1000 UV-Vis Spectrophotometer (Nanodrop Technologies, Wilmington).

### 2.2 Growth and cultivation of bacteria

*Aggregatibacter actinomycetemcomitans* (DSM 11123, German Collection of Microorganisms and Cell Cultures, Braunschweig, Germany) were cultured on fastidious anaerobe agar (FAA) plates (LabM, Heywood, UK), supplemented with 5% sheep blood under anaerobic conditions (80% N_2_, 10% H_2_, 10% CO_2_) for 48 h at 37°C. A few colonies of *A. actinomycetemcomitans* from the FAA plate were inoculated overnight into a brain heart infusion medium (BHI; Oxoid, Wesl, Germany) supplemented with 10 μg/mL vitamin K (Roth, Karlsruhe, Germany) under anaerobic conditions (Kommerein et al., 2017). The overnight cultures were adjusted to OD_600_ = 0.1 in BHI and used for *in vitro* cell culture assays and infections in the mouse model.

### 2.3 *In vitro* cell culture assays

Murine macrophages (RAW 264.7) were used to measure antibacterial activities and IFN-β expression under *in vitro* conditions. Macrophages were provided by Clinic for Pediatric Pneumology, Allergology and Neonatology, Hannover Medical School, Germany. Macrophages were cultured in Roswell Park Memorial Institute (RPMI) 1,640 medium (PAN-Biotech GmbH, Germany) with 10% Fetal calf serum (FCS) (P30-3309, PAN-Biotech GmbH, Germany) and 1% Penicillin/Streptomycin (A2212, Biochrom GmbH, Germany). Macrophages (1 × 10^5^ cells) were grown with 1.5, 3.25, and 6. 25 μg/mL of silver nitrate and tetracycline at same concentrations under standard cell culture conditions to determine the cytotoxicity. Followed by 48 h of incubation, the cells were treated with (1:10) cell counting kit (WST-8/CCK 8) (ab228554) for 2 hours, and then absorbance at 460 nm was measured using a multimode plate reader (Tecan, Infinite M200Pro, Männedof, Switzerland). Macrophages (1 × 10^5^ cells per ml) were seeded on implant surfaces with silver (1.5 µg/disc), tetracycline (1.5 µg/disc) and titanium surfaces. After 24 h, macrophages were infected with 5 µL of *A. actinomycetemcomitans* at OD_600 nm_ = 0.1, approximately corresponding to 5.77 × 10^6^ CFU/mL suspended in PBS. At 6 and 24 h after the infection, cell culture supernatants were collected to count bacteria after diluting supernatants (1:10) in PBS. 100 μL from each dilution was streaked on FAA agar plates with 5% sheep blood, and plates were incubated for 48 h at 37°C with 5% CO_2_. The expression of IFN-β in cell culture supernatants was measured with Verikine-HS Mouse IFN-β ELISA Kit (PBL Assay Science) following the manufacturer’s instructions. Cells were stained with a Calcein AM and Propidium iodide (diluted 1:1,000 in PBS) at room temperature for 30 min and imaged with a confocal laser scanning microscope (CLSM) (SP-8, Leica Microsystems, Wetzlar, Germany). Propidium iodide and Calcein AM signals were measured with a multi-wavelength argon laser (excitation wavelength 488 nm) and an emission range of 500–550 nm (Doll et al., 2019; Rahim et al., 2021).

### 2.4 Analysis of mRNA expression level by RT-qPCR

Macrophages (1× 10^5^ cells/mL) seeded on the surface of titanium and titanium surfaces with silver or tetracycline were cultured for 24 h in a cell culture incubator. After 24 h, macrophages were kept either sterile or infected with *A. actinomycetemcomitans* and cultured further for 24 h. Followed by 48 h of incubation, cells were removed from the implant surfaces using a cell scrapper, and RNA from these cells was extracted using NucleoSpin RNA Isolation Kit (Macherey-Nagel, Germany) following the manufacturer’s protocol. The concentration of isolated RNA was measured using Nanodrop ND-1000 UV-Vis Spectrophotometer (Nanodrop Technologies, Wilmington). RNA was reverse transcribed into cDNA using the Prime-Script First Strand cDNA Synthesis Kit (TaKaRa, Kyoto, Japan) according to the provided protocol. Primers and SYBR Green (Bioline, London, UK) were added to the cDNA, and quantitative real-time PCR (RT-qPCR) was performed (Supplementary Table S1). All samples were measured as triplicates and PCR reactions were completed in a LightCycler 480 (Roche, Basel, Switzerland). The 2^−ΔΔCT^ method was used to measure the relative mRNA expression of genes, using β-actin as the housekeeping gene. Primer sequences of genes are listed in Supplementary Table S1.

### 2.5 *In vivo* implantation and infection

Animal experiments were performed with permission number: 33.12-42502-04-17/2580 from the Lower Saxony State Office for Consumer Protection and Food Safety, Germany, on female transgenic IFN-β reporter mice strain C. Balb/c1-Ifnb1tm1.2Lien (Lienenklaus et al., 2009). Animals were bred at the Central Animal Facility, Hannover Medical School, Germany, and kept under standard conditions with optimum food and water supply. Animal experiments were conducted by following instructions from ARRIVE guidelines. For implantation, animals were first anesthetized under a sterile hood by intraperitoneal injection of 10 mg/kg ketamine (Albrecht, Germany) and 4 mg/kg xylazine (Rompun, Bayer). The implantation area on the dorsal side of mice was shaved with a hair trimmer (Aesculap Suhl, GmbH, Germany) and then disinfected with 70% ethanol. A surgical pouch was created in this region with surgical scissors and tissue forceps (Fine Science Tools, GmbH, Heidelberg, Germany). Titanium implants were gently inserted into these pouches, and wounds were closed with a simple interrupted suture (Ethicon Vicryl, Johnson & Johnson Medical GmbH). Within an hour after implantation, 5 μL of *A. actinomycetemcomitans* (OD_600 nm_ = 0.1, approximately corresponding to 5.77 × 10^6^ CFU/mL) suspended in PBS were injected directly into these implants. Before imaging, D-luciferin (150 mg/kg) (PerkinElmer) diluted in Dulbecco’s Phosphate Buffered Saline (PBS; Biochrom GmbH, Berlin, Germany) was intraperitoneally injected into these animals. After 15 min, animals were anesthetized with an XGI-8 gas anesthesia unit (PerkinElmer) using 2% isoflurane, and bioluminescence was recorded with an *in vivo* imaging system (IVIS Spectrum CT, PerkinElmer). The bioluminescence was processed using Living Image Software Version 4.5 (PerkinElmer).

### 2.6 Colony forming unit assay, sanger sequencing and histology

Followed by 3 weeks of implantation, mice were euthanized, and subcutaneous implants were explanted and added directly into BHI with vitamin K. These implants were homogenized with a tissue homogenizer (Precellys^®^24 Tissue Homogenizer–Bertin Instruments). The homogenized bacterial mixture was ten-fold serially diluted in PBS, and then 100 µL from each dilution was streaked on FAA plates with 5% sheep blood. FAA plates were incubated at 37°C for 48 h with 5% CO_2_, and then visible bacterial colonies were counted on the plates. To confirm that the colonies observed on plates belonged to injected *A. actinomycetemcomitans*, bacteria from colonies were mixed with water and heated at 95°C for 15 min, and 16S rRNA gene was amplified, sequenced and identified by comparison with the known 16S rRNA sequences (da Silva et al., 2014; Schaumann et al., 2014). For histology, peri-implant tissues were fixed for 2 days at room temperature in 3.5% buffered formalin (Otto Fischer, Saarbrücken, Germany). Tissue specimens were embedded in paraffin using an automated embedding system (Pathcentre Tissue Processor, Shandon, Dreieich, Germany) and cut into 5 µm thin sections with Leica RM 2155 microtome, mounted on poly-L-lysine coated glass slides. Thin sections were dried for 24 h at 37°C. Before staining, tissue sections were deparaffinized in xylene (3 × 10 min) and rehydrated in a series of decreasing concentrations of alcohol. Tissue sections were first rinsed in distilled water for 30 min, and then stained with Mayer’s hematoxylin (Merck, Darmstadt, Germany), rinsed again in tap water for 10 min, then stained with 1% eosin (Merck) for 30 min. Tissues were dehydrated in graded concentration series of ethanol and mounted in Eukitt (Labonord, Mönchengladbach, Germany) according to established protocol (Diekmann et al., 2016). Stained tissues were imaged with a Zeiss Axioscope 40 microscope combined with a Zeiss AxioCam Mrc digital Camera and Zeiss AxioVision software (Zeiss, Oberkochen, Germany).

### 2.7 Statistical analysis

Statistics were calculated in GraphPadPrism v. 8. The Shapiro-Wilk-test was used to assess normality unless indicated otherwise in the figure legends. Equality of variances was assessed graphically based on both data dot-plots and homoscedasticity plots. Where multiple comparison correction is applicable, family-wise *p*-values are reported. Symbols ****, ***, **, and * indicates a family-wise probability of *p* ˂ 0.0001, *p* ˂ 0.0010, *p* ˂ 0.010, and *p* ˂ 0.050.

## 3 Results

### 3.1 Analysis of silver and tetracycline on titanium for *in vivo* implantation

**FIGURE 1:**
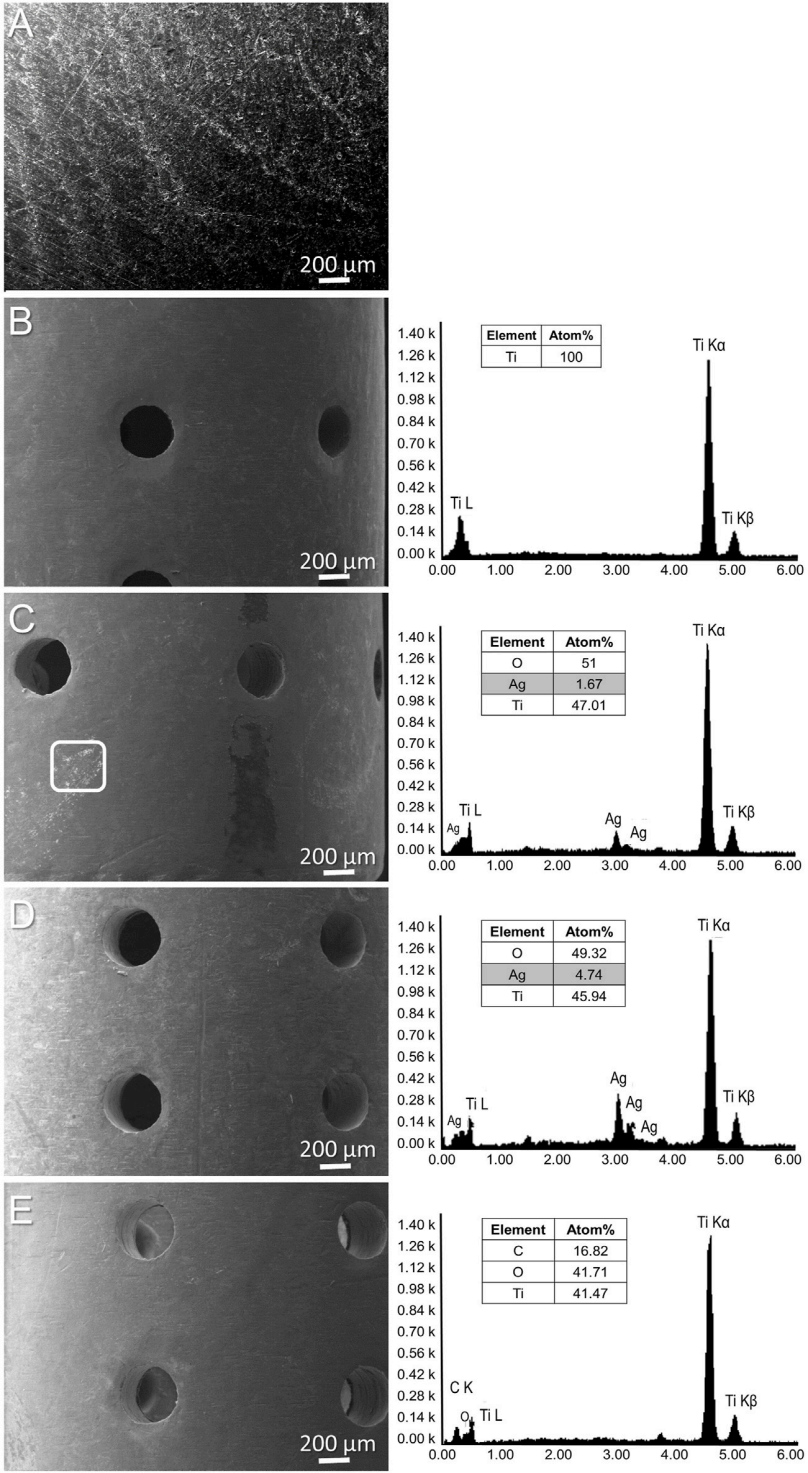
SEM of titanium surface **(A)**. SEM and EDS (left side) analysis of porous titanium implant **(B)**, cylindrical porous titanium implants with 500 μg/mL of Silver **(C)**, 1 mg/mL of Silver **(D)**, and titanium implant with tetracycline **(E)**.

For *in vitro* evaluation, silver and tetracycline were added to the surface of titanium discs. Scanning electron microscopy (SEM) analysis could not show the presence of a silver layer on titanium discs due to the higher surface roughness, surface area of implants and lower concentration of added silver (Figure 1A). For *in vivo* implantations, cylindrical porous titanium implants were selected so that injected bacteria have the possibility to establish biofilms even in the presence of host immune cells. Silver and tetracycline were deposited on cylindrical implants using simple heat evaporation method so that these antibacterial agents can immediately interact with adjacent tissues (Supplementary Figure S1). The morphology and composition of silver and tetracycline on implants were observed using a scanning electron microscope (SEM) and Energy dispersive spectroscopy (EDS). SEM and EDX analyses of titanium before adding silver or tetracycline (Figure 1B). SEM analysis revealed the presence of silver around cylindrical implants (Figure 1C, White Square). Energy dispersive spectroscopy (EDS) analysis confirmed the presence of silver on the surface of titanium (Figures 1C, D). EDS analysis of titanium with tetracycline showed the presence of carbon, oxygen and titanium (Figure 1E). The absorbance assay confirmed that the silver and tetracycline on implants released within 24 h after incubation with liquid medium (Supplementary Figure S2). SEM, EDS, and absorbance assays confirmed that porous titanium implants had silver on the surfaces of titanium.

### 3.2 IFN-β expression and antibacterial activity inside murine macrophages

**FIGURE 2:**
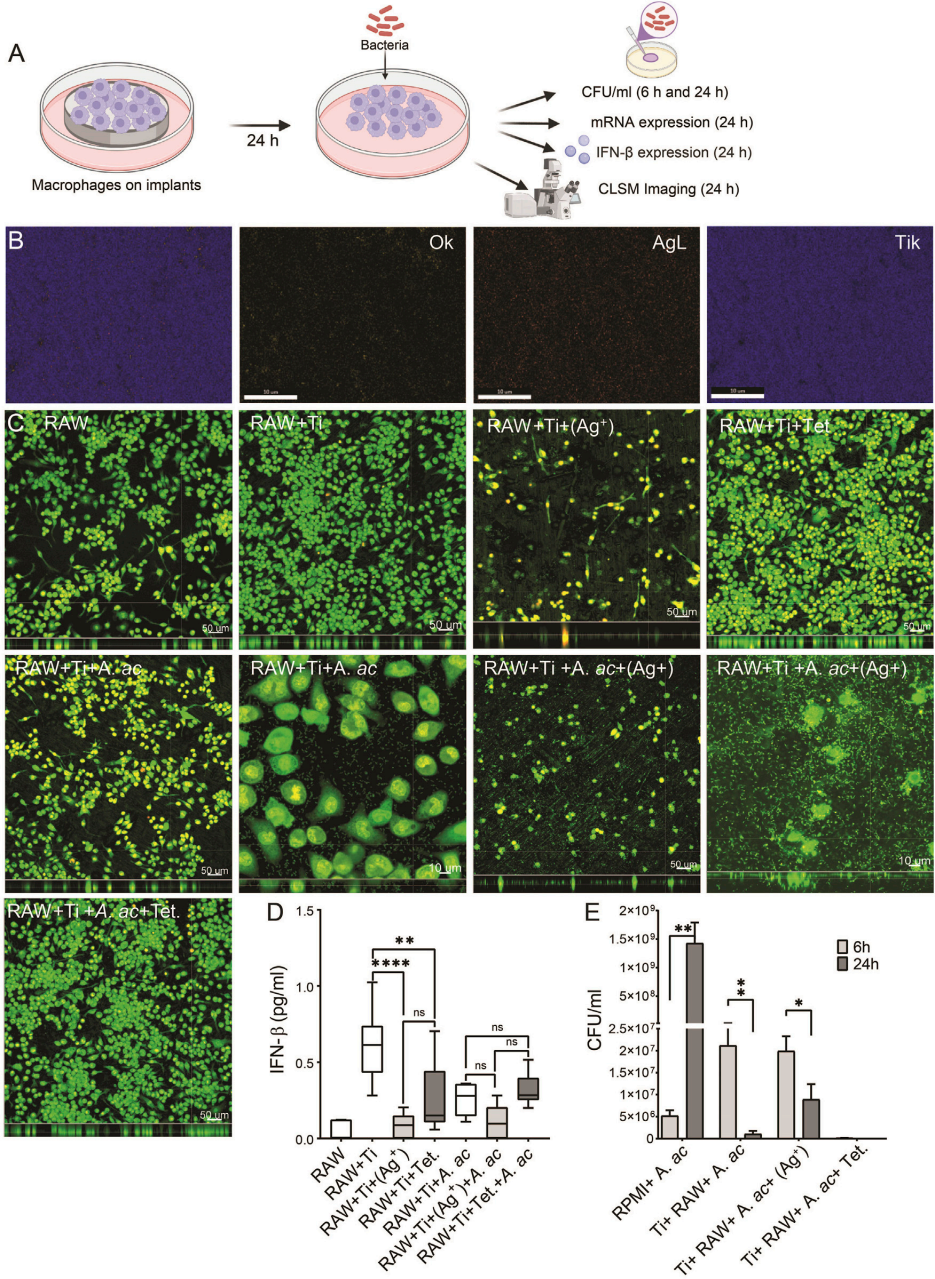
Compatibility and IFN-β expression inside macrophages. **(A)** Schematic diagram explaining that macrophages (1 × 10^5^ cells per ml) were seeded on titanium, titanium with silver and titanium with tetracycline. After 24 h of incubation, cells were infected with *A. actinomycetemcomitans*, and at 6 and 24 h after infection, antibacterial activity, mRNA level and IFN-β expression were measured. **(B)** mapping element on titanium surface with silver corresponding to oxgen (OK), silver (AgL) and titanium (TiK). **(C)** Macrophages are shown as green fluorescence on cell culture plate (RAW), on titanium (RAW + Ti), on titanium with silver [RAW + Ti+(Ag^+^)], titanium with tetracycline (RAW + Ti + Tet.), titanium with *A. actinomycetemcomitans* (RAW + Ti + *A. ac*), titanium surfaces with silver and *A. actinomycetemcomitans* [RAW + Ti + *A. ac*+(Ag^+^)], titanium surfaces with tetracycline with *A. actinomycetemcomitans* (RAW + Ti + Tet.+*A. ac*). **(D)** Box plot showing IFN-β expression in the cell culture supernatants collected from macrophages labelled according to information as mentioned before. These results are the average ± standard errors of means of six samples from two experiments. The samples containing titanium were statistically compared with GraphPad prism using one-way ANOVA followed by Tukey’s multiple comparisons test (*n* = 6 per group). Symbols ****, **, and * indicates a family-wise probability of *p* <0.0001, *p* <0.010, and *p* <0.050. **(E)** The growth of *A. actinomycetemcomitans* in cell culture medium (RMPI + *A. ac*), in supernatants of macrophages cultured on titanium (Ti+ RAW+ *A. ac*), titanium with silver [Ti + RAW + *A. ac*+(Ag^+^)] and titanium with tetracycline (Ti + RAW + *A. ac* + Tet.) at 6 (light grey) and 24 h (dark grey) after infection. Multiple *t*-test was used for statistical analysis. Symbols **, and * indicates a family-wise probability of *p* < 0.010, and *p* < 0.050.

Macrophages were grown with various concentrations of silver nitrate and tetracycline for 48 h, and then the viability of cells was measured. Macrophages could not survive with a concentration of silver nitrate higher than 1.5 μg/mL (Supplementary Figure S3). Therefore, 1.5 μg/mL of silver nitrate was applied on titanium surfaces. Macrophages were seeded on titanium and titanium with silver or tetracycline for 24 h and then infected with *A. actinomycetemcomitans*. Cell culture supernatants were collected at 6 and 24 h after infection to count bacteria and measure IFN-β expression (Figure 2A). Energy dispersive spectroscopy (EDS) detected a minor layer of silver on the surface of titanium (Figure 2B, AgL). Macrophages cultured under sterile conditions were compatible with cell culture plates and titanium (Figure 2C, RAW and RAW + Ti). The compatibility of macrophages was reduced on titanium surfaces with silver [Figure 2C, RAW + Ti+(Ag^+^)]. Macrophages were compatible on titanium surfaces with tetracycline (Figure 2C, RAW + Ti + Tet.). The presence of *A. actinomycetemcomitans* did not influence the growth and morphology of macrophages on titanium (Figure 2C, RAW + Ti + *A. ac*). The compatibility of macrophages decreased on titanium with silver in the presence of *A. actinomycetemcomitans* [Figure 2C, RAW + Ti + *A. ac+* (Ag^+^)]. *A. actinomycetemcomitans* were visible on plain titanium surfaces and titanium surfaces with silver [Figure 2C, white arrows on RAW + Ti + *A. ac* and RAW + Ti + *A. ac+* (Ag^+^)]. Infected macrophages showed compatibility on titanium surfaces with tetracycline, and bacteria were not microscopically visible (Figure 2C, RAW + Ti + Tet. *A. ac*). There was IFN-β expression inside macrophages cultured on cell culture plates and titanium (Figure 2D). The IFN-β expression inside the macrophages seeded on titanium with silver significantly decreased compared to cells cultured on titanium (Figure 2D). The IFN-β expression inside macrophages grown on titanium with tetracycline was non-significantly higher than in cells cultured on titanium with silver. Presence of *A. actinomycetemcomitans* could not stimulate higher IFN-β expression inside cells because of the strong phagocytic activities of macrophages (Figure 2D). The IFN-β expression inside infected macrophages cultured on titanium with silver non-significantly decreased compared to infected titanium (Figure 2D). The IFN-β expression inside infected macrophages cultured on titanium with tetracycline non-significantly increased compared to cells cultured on infected titanium and titanium with the silver (Figure 2D). *A. actinomycetemcomitans* showed the highest growth in cell culture medium after 24 h of incubation (Figure 2E). Interestingly, macrophages cultured on titanium showed strong antibacterial effects, even higher than cells cultured on titanium with silver (Figure 2E). Antibacterial activities of implants with tetracycline on their surfaces were the highest, and bacteria were not detectable in their supernatants after 6 h of infection (Figure 2E; Supplementary Figure S4). Overall, results from *in vitro* assay showed that titanium surfaces with silver strongly influenced the compatibility, IFN-β production and antibacterial activities.

### 3.3 The mRNA expression level of IFN-β, interferon-stimulated genes and pro-inflammatory cytokines inside macrophages

**FIGURE 3:**
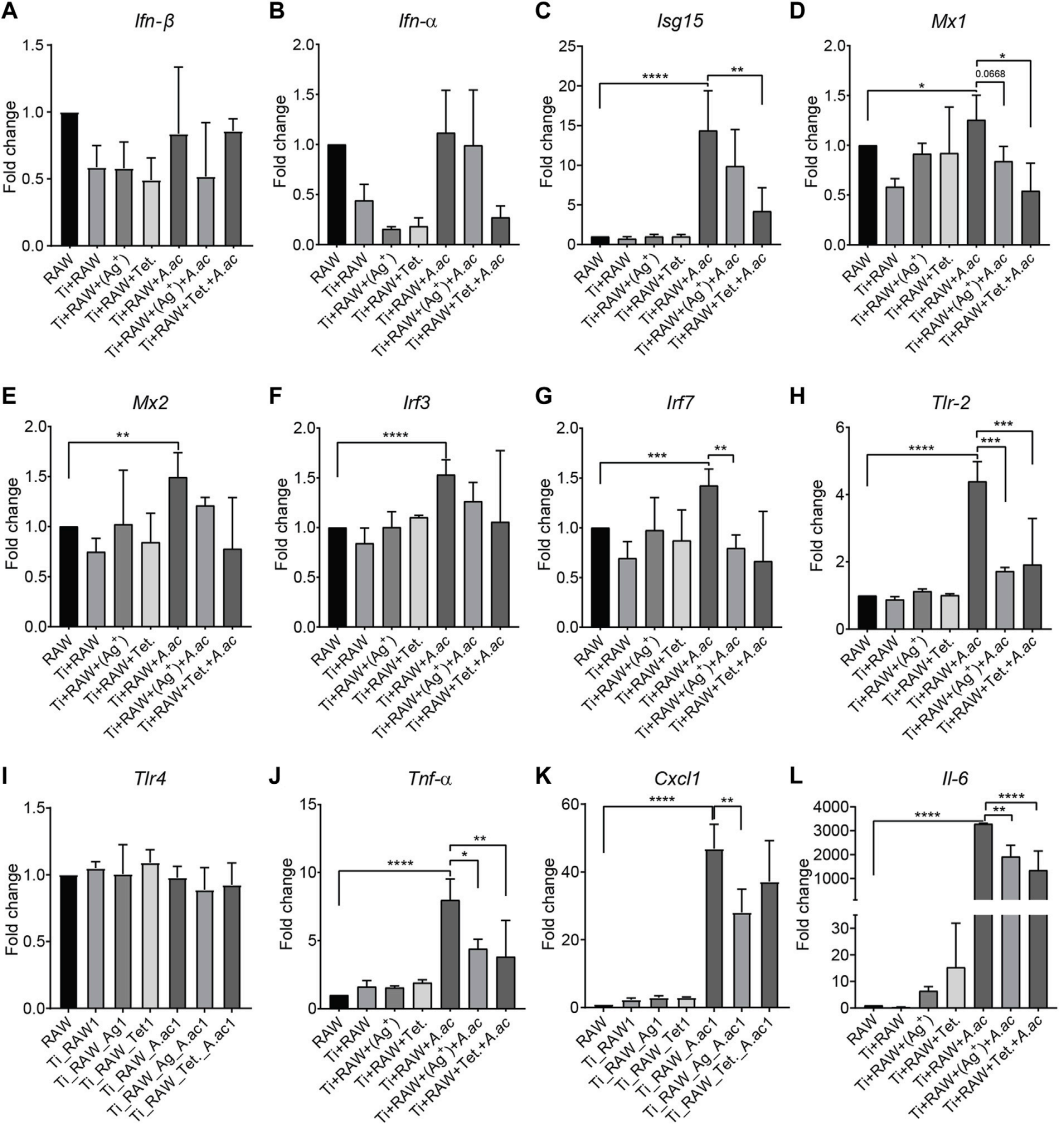
The mRNA expression level of IFN-β, interferon-stimulated genes and proinflammatory cytokines inside murine macrophages. Murine macrophages (RAW) were cultured on titanium (RAW + Ti), titanium with silver [RAW + Ti+(Ag^+^)], titanium with tetracycline (RAW + Ti + Tet.). After 24 h of incubation, *A. actinomycetemcomitans* was added into cells cultured on titanium (RAW + Ti + *A. ac*), titanium with silver [RAW + Ti + *A. ac*+(Ag^+^)], titanium with tetracycline (RAW + Ti + Tet.+*A. ac*). Following 24 h of infection, mRNA expression levels of the *Ifn-β, Ifn-α*, *Isg15*, *Mx1*, *Mx2*, *Irf3*, *Irf7, Tlr-2*, *Tlr-4, Tnf-α, Cxcl-1*, and *Il-6* were measured by RT-qPCR **(A–L)**. The expression of genes was measured two times, and the data shown represents a single experiment. Bars depict mean ± SD. Statistical analysis was performed with a Graph pad prism by using the One-Way ANOVA followed by Tukey’s test. Symbols ****, ***, **, and * indicates a family-wise probability of *p* < 0.0001, *p* < 0.0010, *p* < 0.010, and *p* < 0.050. Legends: *Ifn-β, interferon-beta; Ifn-α, interferon-alpha; Isg15, Ifn-stimulated gene 15; Mx1, The murine myxovirus resistance 1; Mx2, The murine myxovirus resistance 1; Irf3, IFN regulatory factor 3; Irf7, IFN regulatory factor 7; Tlr2, Toll-like receptor 2; Tlr4, Toll-like receptor 4; Tnf-α, Tumor necrosis factor alpha; Cxcl1, CXC motif chemokine ligand 1; Il-6, Interleukin 6.*

To investigate the effect of titanium surfaces with silver or tetracycline on *Ifn-β* and interferon-stimulated genes, the mRNA expression level of *Ifn-β*, interferon-stimulated genes and pro-inflammatory cytokines were measured using RT-qPCR. The mRNA expression levels of *Ifn-β*, *Isg15*, *Mx1*, *Mx2*, *Irf3*, *Irf7, Tlr-2*, *Tlr-4, Tnf-α, Cxcl-1,* and *Il-6* genes inside macrophages that were cultured on titanium and titanium with silver and tetracycline remained similar in sterile conditions (Figures 3A–L). Upon infection, the mRNA expression level of *Ifn-β* inside macrophages did not show any upregulation. A non-significant downregulation in mRNA expression level of *Ifn-β* was observed inside cells cultured on titanium with silver compared to titanium and titanium with tetracycline (Figure 3A). Of note, the infection caused significant upregulation in mRNA expression level of interferon-stimulated genes (*Isg15*, *Mx1*, *Mx2*, *Irf3*, and *Irf7*) and pro-inflammatory cytokines (*Tnf-α, Cxcl-1,* and *Il-6* genes) (Figures 3C–G, J–L). In the presence of silver, the mRNA expression levels of *Ifn-β*, *Isg15*, *Mx2*, and *Irf3* considerably decreased while the expression levels of *Mx1*, *Irf7, Tlr-2*, *Tnf-α, Cxcl-1,* and *Il-6* genes significantly decreased inside infected macrophages (Figure 3). The mRNA expression level of Tlr-2 increased significantly upon infection with *A. actinomycetemcomitans* confirming the involvement of this gene in detecting this bacterium (Figure 3H). In the presence of silver and tetracycline, there was a significant decrease in the expression of Tlr-2 inside macrophages compared to titanium. Notably, the mRNA expression levels of *Ifn-α*, *Isg15*, *Mx1*, *Mx2*, *Irf7, Tlr-2*, *Tnf-α, Cxcl-1,* and *Il-6* downregulated inside the macrophages cultured on titanium with tetracycline. Although infection did not influence mRNA expression level of *Ifn-β* gene inside macrophages cultured on titanium surfaces and titanium with silver or tetracycline, the expression levels of *Tnf-α, Cxcl-1* and *Il-6* genes significantly increased.

### 3.4 Subcutaneous implants with silver decrease the expression of IFN-β in mice

**FIGURE 4:**
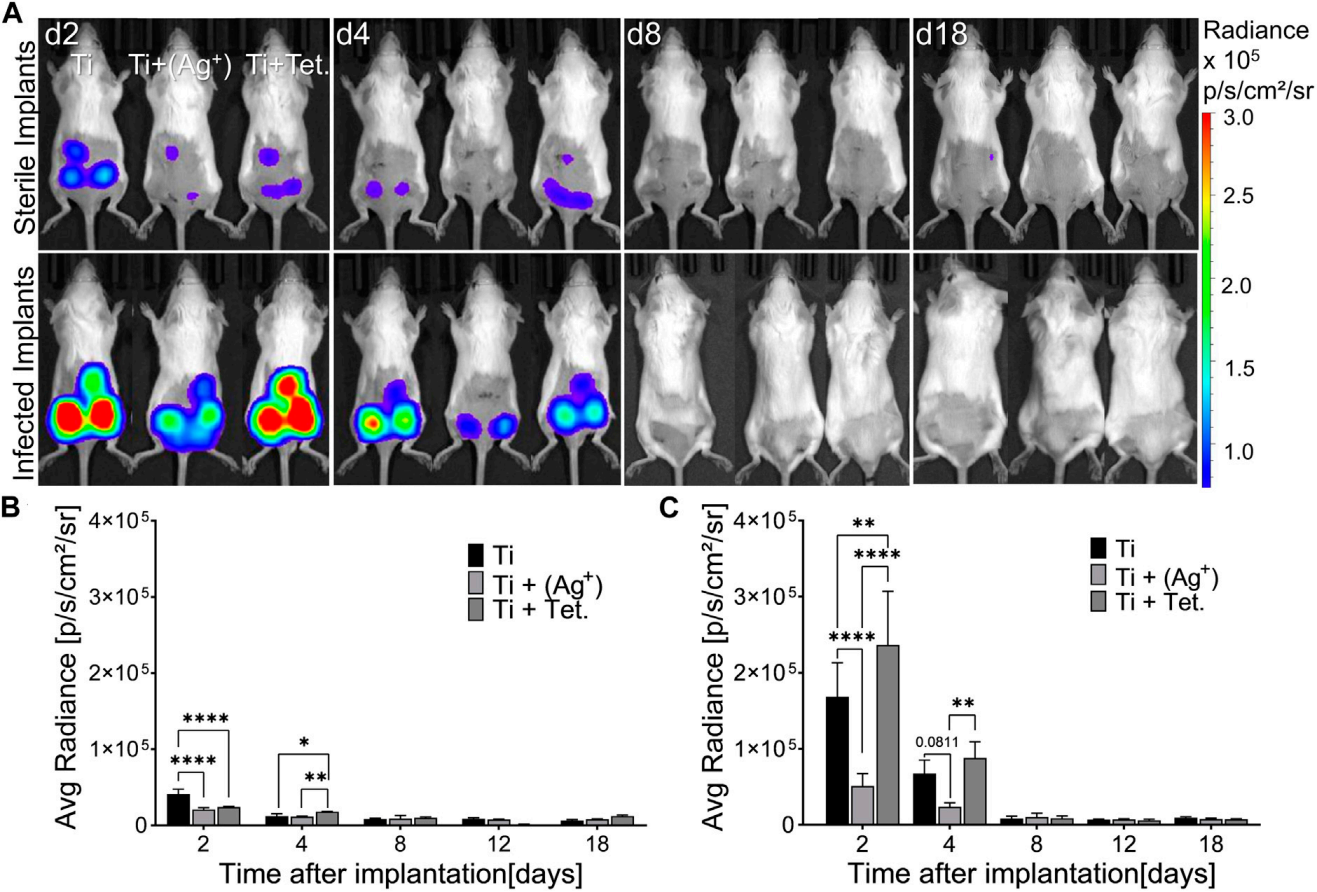
Implant surfaces with silver decrease IFN-β expression in mice. Cylindrical porous titanium implants (Ti), titanium with silver [Ti + (Ag^+^)], and titanium with tetracycline (Ti + Tet.) were implanted subcutaneously in transgenic mice. Implants were kept either sterile or infected with *A. actinomycetemcomitans* (Infected implants). Images of the luminescence representing IFN-β expression were recorded by *In Vivo* Imaging System at the indicated time points **(A)**. Pseudo colors indicate the intensity of luminescence according to the scale on the right side. A Graphical representation of IFN-β shows average radiance over the time course in mice bearing sterile implants **(B)** or in mice infected with *A. actinomycetemcomitans* **(C)**. Error bars represent standard deviations. Statistical analysis was performed with a Graph pad prism by using the Two-Way ANOVA test for multiple comparisons followed by Tukey’s multiple comparison test. Symbols ****, **, and * indicates a family-wise probability of *p* < 0.0001, *p* < 0.010, and *p* < 0.050. These results are representative data from one of the three experiments.

The expression of IFN-β in transgenic mice was measured using an advanced non-invasive *in vivo* imaging system (IVIS). There was no immediate induction of IFN-β expression in the early hours after implantation and infections. Followed by 2 days, it was possible to measure IFN-β expression (Figure 4). The variation in the induction of IFN-β expression around titanium and titanium with silver under sterile conditions was clearly visible on subcutaneous implants (Figure 4A, d2, sterile implants). The luminescent signal representing IFN-β expression was lower around subcutaneous titanium with silver and tetracycline compared to titanium implants (Figure 4A, d2, sterile implants). Infection with *A. actinomycetemcomitans* induced higher expression of IFN-β around subcutaneous implants compared to sterile implants (Figure 4A, infected implants). The expression of IFN-β around subcutaneous implants with silver decreased significantly compared to titanium implants (Figure 4A, d2, Ti + Ag). Interestingly, the expression of IFN-β around infected implants with tetracycline increased and was significantly higher than infected implants with silver and plain titanium implants (Figures 4A, C, d2, Ti +Tet.). On day 4, the expression of IFN-β decreased around titanium, titanium with silver, and tetracycline compared to the expression measured on day 2 in both sterile and infected mice (Figures 4A–C, d4). IFN-β expression on day 4 decreased significantly around subcutaneous implants with silver compared to titanium and titanium with tetracycline in the infected group (Figure 4A, C, d4). From day 8 to day 18, the non-invasive *in vivo* imaging system could not capture the expression of IFN-β around subcutaneous implants within live animals (Figure 4A, d8-18). During the observation period, all animals stayed healthy and maintained their body weights, and there were no signs of weight loss or decreased food or water consumption (Supplementary Figure S5). In conclusion, the *in vivo* analysis of reporter mice having a complicated immune system confirmed that the presence of silver on implant surface significantly decreased IFN-β expression under sterile and infected conditions.

### 3.5 Histological analysis of the soft-tissue interfaces around subcutaneous implants

**FIGURE 5:**
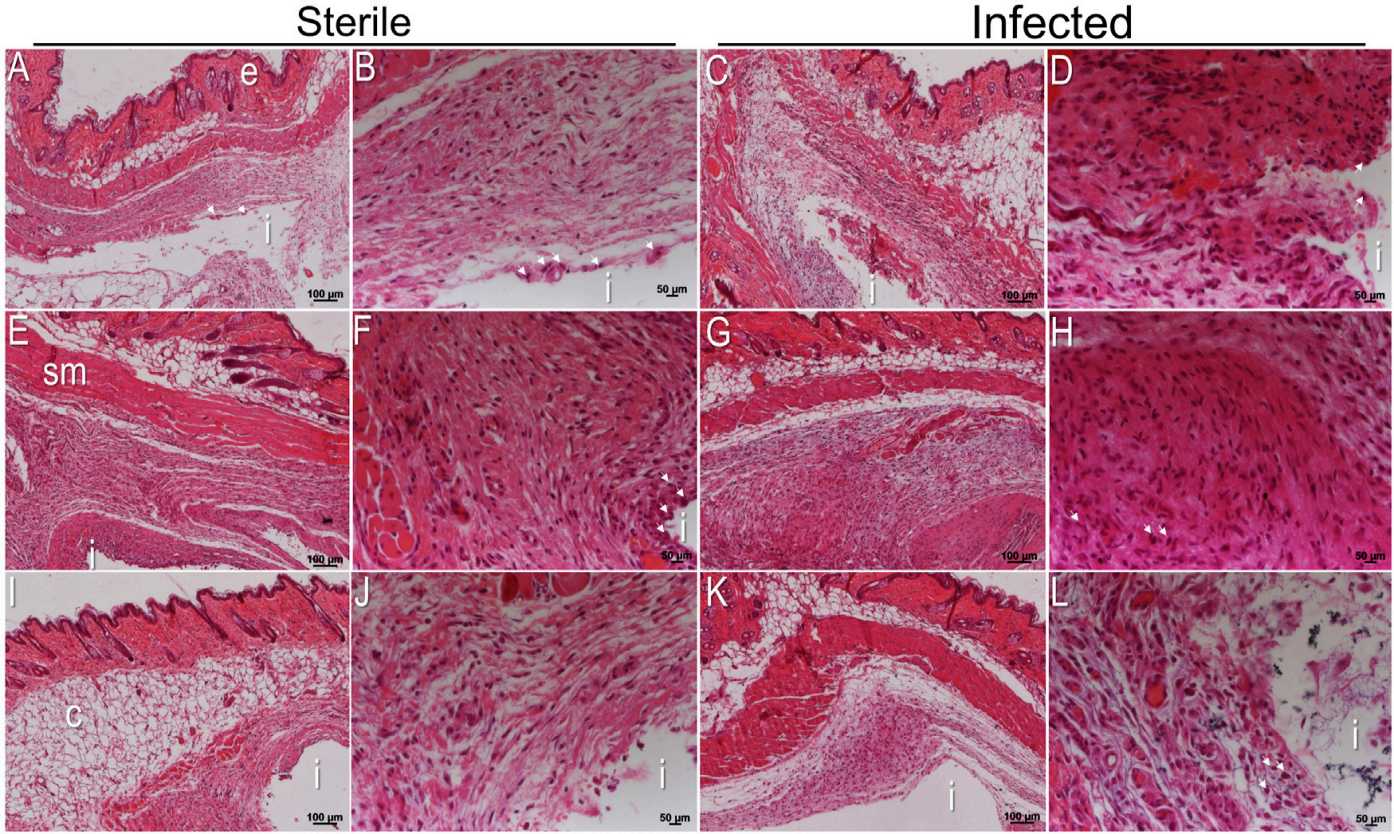
Histological analysis of tissue sections after 3 weeks of implantation. H&E stained sections of the peri-implant tissue interface near sterile titanium **(A,B)**, titanium with silver **(E,F)**, and titanium with tetracycline **(I,J)**. H&E stained sections of *A. actinomycetemcomitans* infected peri-implant tissue interface near titanium **(C,D)**, titanium with silver **(G,H)**, and titanium implants with tetracycline **(K,L)**. Each H&E stained section is shown at two different magnifications. Scale bars of each peri-implant tissue are shown at 100 μm and 50 µm. (i) Indicates the site of implantation in each image.

After 3 weeks of implantation, the status of the peri-implant tissue interfaces around subcutaneous implants was monitored with hematoxylin and eosin (H&E) staining. The histological analysis indicated that tissues surrounding all implants consisted mainly of intervening blood vessels, connective tissue, adipose tissue, and muscle fibers (Figures 5A–L). Around sterile titanium implants, there was mild recruitment of host inflammatory cells expected as a foreign body response (Figures 5A, B, white arrows). Notably, the infiltration of host inflammatory cells was higher at the peri-implant tissue interface adjacent to implants with silver than in titanium implants (Figures 5E, F, white arrows). The recruitment of inflammatory cells around implants with tetracycline was similar to titanium implants (Figures 5I, J). A prominent cellular response with a thick interface was detected inside tissues surrounding infected implants. There were more inflammatory cells at the tissue implant interfaces compared to sterile implants (Figures 5C, D). The infected peri-implant tissues adjacent to implants with silver showed a significantly higher density of inflammatory cells indicative of high inflammation than tissue from sterile titanium implants and infected titanium with tetracycline (Figures 5G, H; Supplementary Figure S6). Except for the cellular infiltration, there was no fibrotic encapsulation in the peri-implant tissues adjacent to implants with silver (Figures 5G, H). The recruitment of immune cells at the peri-implant tissue interface near titanium with tetracycline was similar to titanium (Figures 5K, L). After observing higher recruitment of inflammatory cells in the tissue interfaces of implants with silver, it was decided to monitor implant surfaces with confocal microscopic analysis. Confocal microscopy indicated that it was impossible to detect any differences in the amount of host cells directly on plain titanium and titanium carrying antibacterial agents (Supplementary Figure S7). Overall, histological analysis of peri-implant tissue helped identify the status of inflammatory cells and, importantly, revealed higher recruitment of host immune cells around implants with silver.

### 3.6 Antibacterial activity of subcutaneous implants carrying silver and tetracycline in mice

**FIGURE 6:**
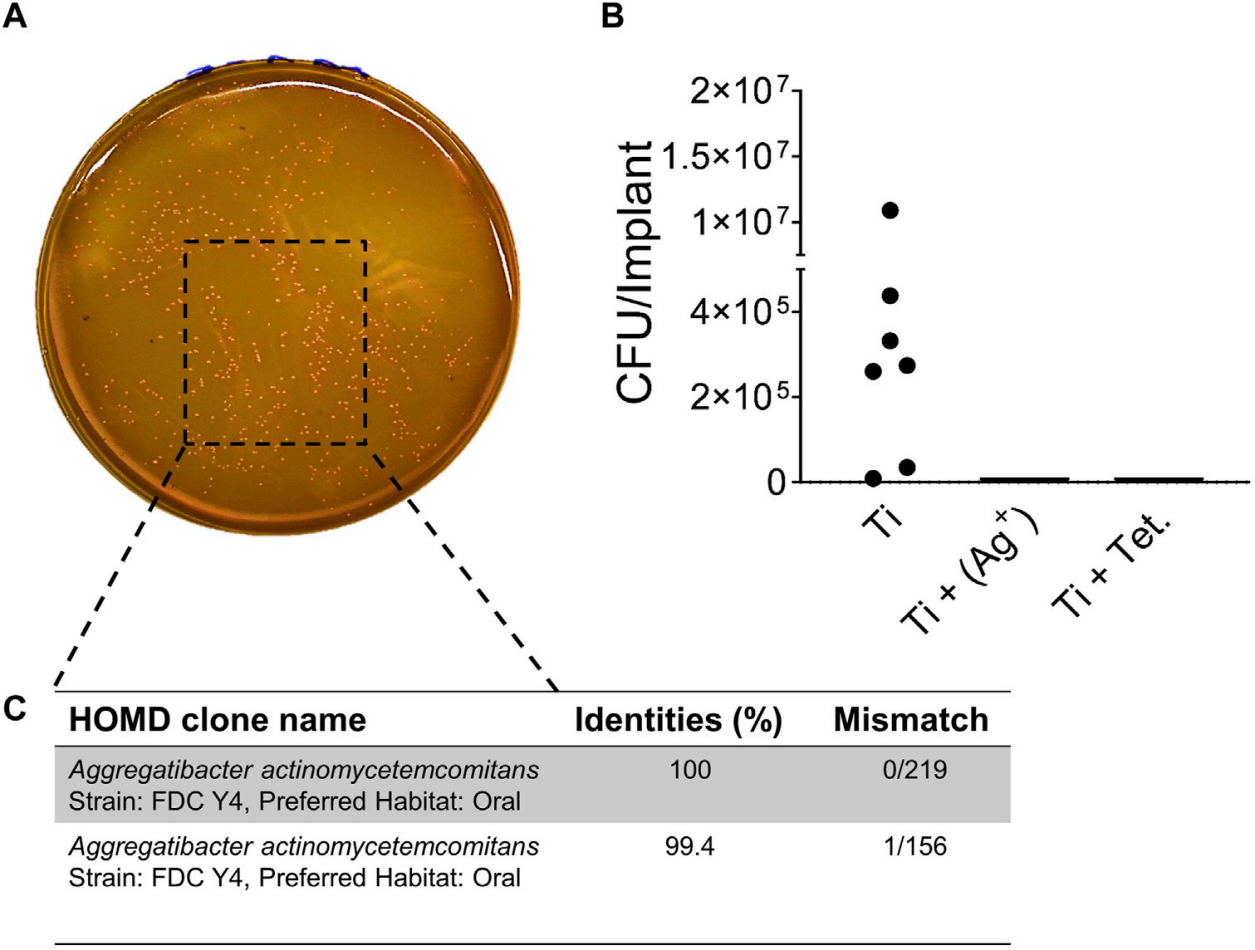
*In vivo* antibacterial activity of implants with silver. *A. actinomycetemcomitans* isolated from titanium implants on FAA plates **(A)**. Colony-forming units per implant inside titanium (Ti) implants **(B)**. Sanger Sequencing confirming *A. actinomycetemcomitans* from titanium implants **(C)**.

After 3 weeks of implantation, subcutaneous implants were isolated from the mice to monitor antibacterial activities. Injected *A. actinomycetemcomitans* were still present inside titanium implants after 3 weeks of implantation (Figures 6A, B). However, it was impossible to quantify injected A. actinomycetemcomitans from explanted titanium with silver or tetracycline after 3 weeks because of the antibacterial effect of silver or tetracycline (Figure 6B). Speculating that the native bacteria from the mice skin could enter into the subcutaneous implants and appear on FAA plates, Sanger sequencing was applied to verify that isolated bacteria from implants. Results of Sanger sequencing confirmed that the bacteria recovered from subcutaneous implants were purely *A. actinomycetemcomitans* (Figure 6C). This analysis confirmed that implants with silver and tetracycline maintained their antibacterial effects. Moreover, cylindrical titanium implants prevented the risk of bacterial contamination from the fur and skin of mice.

## 4 Discussion

The formation of complex bacterial biofilm on medical implants can lead to inflammatory diseases and failure of the implant. Biofilms can elude the host immunity and resist antibiotics. Due to the antibiotic-resistant nature of biofilms, the systemic administration of antibiotics to treat implant-associated infections remains ineffective. Applying antibacterial agents directly on the implant surface has been observed as promising strategy to prevent biofilms without systemic side effects. Biofilms activate the innate immune system and produce type I interferon (McWhirter et al., 2009; Valle et al., 2013b; Peignier and Parker, 2021). Controlled secretion of type I interferon is a critical part of the host immune system. One aspect that has been insufficiently investigated is the expression of IFN-β during biomaterial-associated infections in the presence of implants with antibacterial agents. In the present study, IFN-β expression and the antibacterial activities of implant surfaces with silver or tetracycline were measured inside murine macrophages and mice during sterile and infected situations. *Aggregatibacter actinomycetemcomitans* was used for infection since it is a periodontal pathogen responsible for periodontal inflammatory diseases, especially juvenile periodontitis and peri-implantitis (Meyer and Fives-Taylor, 1997; van Winkelhoff and Wolf, 2000). In addition, in our previous publication, subcutaneous infections of implants with *A. actinomycetemcomitans* in mice elicited strong IFN-β expression compared to other periodontal pathogens (Rahim et al., 2020b). Macrophages were used for *in vitro* evaluations since they are the predominant immune cells at the host-implant interface (Brown et al., 2012). Silver nitrate was applied on the surface of titanium implants since this formulation was expected to demonstrate higher intercellular penetration (Li et al., 2017; Kędziora et al., 2018). The quantitative cytotoxicity assay (CCK-8) showed that a concentration of silver higher than 1.5 μg/mL was toxic for the cells; therefore, 1.5 μg/mL of silver was applied on the surface of titanium implants using heat evaporation method. In agreement with this finding, Silver nanoparticles (AgNPs) at concentrations ≤ 1 μg/mL were non-cytotoxic for human gingival fibroblasts (Ipe et al., 2020). Silver has also been reported to show antibacterial effects against *A. actinomycetemcomitans* (Sardari et al., 2012; Milić et al., 2015; Chi et al., 2018). The presence of silver and tetracycline directly on implant surfaces was expected to have immediate interaction with the adjacent tissue at peri-implant tissue interfaces. Macrophages were well spread on the surface of titanium and emitted green fluorescence that confirmed their high viability. Macrophages produced IFN-β and showed antibacterial activities against *A. actinomycetemcomitans*. On the surface of titanium with silver, there were noticeably lesser cells. The IFN-β production inside cells cultured on implants with silver significantly decreased compared to sterile titanium. Upon infection, the number of macrophages on titanium with silver reduced, and a significant decrease in IFN-β production was observed. Under sterile and infected conditions, macrophages remained compatible with titanium having tetracycline. However, the IFN-β expression inside macrophages cultured on titanium with tetracycline under sterile conditions significantly decreased compared to titanium surfaces. Macrophages cultured on titanium surfaces without antibacterial agents showed phagocytic activities and killed the bacteria after 24 h of their addition. Silver on the surface of titanium decreased the compatibility and phagocytic activities of macrophages compared to titanium with tetracycline; therefore, *A. actinomycetemcomitans* were detectable on surfaces with silver at 6 h after adding bacteria. Titanium and titanium with tetracycline cultured with macrophages showed higher antibacterial activities compared to titanium with silver. To verify the role of molecular mechanisms underlying decreased IFN-β production inside macrophages, mRNA expression levels of *Ifn-β* genes, interferon-associated genes and other pro-inflammatory genes were measured using RT-qPCR. The mRNA expression level of *Ifn-β* genes inside the macrophages cultured on titanium with silver did not show a significant downregulation compared to macrophages used as control under sterile conditions. Similarly, there was no significant downregulation in the mRNA expression level of *Ifn-β* genes inside macrophages cultured on titanium with tetracycline compared to macrophages, which was used as a control under sterile situations. Exposure of macrophages grown on titanium to *A. actinomycetemcomitans* caused a slight upregulation in the mRNA expression level of *Ifn-β* genes. The mRNA expression level of *Ifn-β* genes inside infected macrophages cultured on titanium with silver showed a non-significant downregulation compared to infected macrophages cultured on titanium. The mRNA expression level of *Ifn-β* genes inside infected macrophages grown on titanium with tetracycline did not show a significant downregulation compared to infected macrophages grown on titanium. This finding confirmed that the decrease in IFN-β expression inside macrophages cultured on titanium with silver under sterile or infected conditions was due to the toxic effect of silver on cells. The mRNA expression level of *Ifn-α*, *Isg15*, *Mx1*, *Mx2*, *Irf3*, *Irf7, Tlr-2*, *Tlr-4, Tnf-α, Cxcl-1,* and *Il-6* did not change inside macrophages cultured on all implants under sterile conditions. Infection with *A. actinomycetemcomitans* caused a significant upregulation in the expression levels of *Isg15*, *Mx1*, *Mx2*, *Irf3*, *Irf7, Tlr-2*, *Tnf-α, Cxcl-1,* and *Il-6* genes. Of note, *A. actinomycetemcomitans* caused a significant increase in the expression of Tlr-2, confirming the involvement of this gene inside macrophages in recognizing the bacterium (Gelani et al., 2009). In the presence of silver, the mRNA expression levels of *Mx1*, *Irf7, Tlr-2*, *Tnf-α, Cxcl-1,* and *Il-6* genes significantly downregulated in infected macrophages. Agreeing with these findings, the application of silver nanoparticles in lipopolysaccharide-treated macrophages inhibited the secretion of pro-inflammatory cytokines (TFN-α and IL-6) (David et al., 2014; Tyavambiza et al., 2021). Degradable magnesium alloy with a silver (Mg-Zn-Ag) decreased the interleukin-1α expression in carcinoma cells (Peng et al., 2013). mRNA expression levels of pro-inflammatory genes were also downregulated in the presence of implants with tetracycline. Similarly, the anti-inflammatory effects of tetracycline have been reported by other studies in the literature (Webster and Del Rosso, 2007; Bostanci et al., 2011). *In vitro* analysis inside macrophages convincingly confirmed that investigated antibacterial agents decreased IFN-β expression and the mRNA expression levels of pro-inflammatory *Il-6*, *Tnf-α* and *Cxcl-1* genes. To validate these findings under *in vivo* situations, a reporter mouse model constitutive for IFN-β expression was employed (Lienenklaus et al., 2009). These reporter mice were previously used to monitor IFN-β expression during biomaterial-associated infections by *Pseudomonas aeruginosa* and *A. actinomycetemcomitans* (Rahim et al., 2017a; Rahim et al., 2020b). For *in vivo* implantations, titanium implants in cylindrical porous morphologies were designed to support prolonged biofilm formation even in the presence of host immune cells (Rahim et al., 2020b). Cylindrical porous titanium implants with silver or tetracycline were inserted into the subcutaneous regions on the dorsal sides of mice. A non-invasive *in vivo* imaging system (IVIS) was used to monitor IFN-β expression around subcutaneous implants (Rahim et al., 2020a). IFN-β expression around the subcutaneous implants with silver and tetracycline significantly decreased compared to titanium implants in the sterile group of mice. Infection with *A. actinomycetemcomitans* stimulated higher IFN-β expression around subcutaneous implants compared to the sterile implants. Notably, IFN-β expression around infected subcutaneous implants with silver significantly decreased compared to titanium implants. Agreeing with these findings, using silver nanoparticles in mice decreased IFN-γ expression (Wong et al., 2009). Likewise, nano-silver showed an anti-inflammatory effect by decreasing the production of IFN-γ and infiltration of inflammatory cells in a mouse model (Shin et al., 2018). The administration of silver nanoparticles (Ag-NP) in rats decreased interferon-γ, IL-10, IL-6, IL-10, and TNF-α production (De Jong et al., 2013). It has been reported that administration of tetracycline shows anti-inflammatory activities inside the body and therefore has been used in treating periodontal diseases (Tilakaratne and Soory, 2014; Madi et al., 2018). The expression of IFN-β around infected subcutaneous implants with tetracycline increased compared to implants with silver and titanium implants. An explanation for such an increase in IFN-β expression could be because of the interaction of bacterial products secreted from killed bacteria with a complicated immune system at the peri-implant tissue interface. The histological evaluations of the peri-implant tissue around subcutaneous implants with silver showed higher infiltration of host inflammatory cells for both sterile and infected tissues. Similar to this observation, a study investigating the inflammatory response of silver nanoparticles (AgNPs) in a rat model found that silver ions induced an inflammatory response and triggered higher recruitment of inflammatory cells in the lung tissue (Li et al., 2021). IFN-I is mostly associated with host-beneficial, antibacterial activities: however, recent data have described the potentially detrimental effect of this cytokine (Mizraji et al., 2017). Observing that implant surfaces with silver decreased IFN-β expression inside macrophages and mice, applying silver could be an advantageous therapeutic possibility to control potentially detrimental activities of type I interferon.

## 5 Conclusion

The inflammation, in part regulated by type I interferons, is a crucial component of host immunity. The present study, for the first time, found that implant surfaces with silver decrease the expression of IFN-β inside murine macrophages and mice. Implant surfaces with tetracycline decreased IFN-β expression under *in vitro* conditions, but IFN-β expression increased around these implants in the presence of biofilms inside mice. Overall, implants surfaces with silver, reported to have strong antibacterial effects even against resistant biofilm-forming strains, decrease IFN-β expression, a critical part of the host immune system. Nevertheless, the finding that silver decreases IFN-β expression could be used to control side effects of elevated IFN-β expression, such as impaired immunity with tissue damage in inflammatory diseases.

## Data Availability

The original contributions presented in the study are included in the article/Supplementary Material, further inquiries can be directed to the corresponding author.
