# Supplementary material for: Effect of titanium implants along with silver ions and tetracycline on type I interferon-beta expression during implant-related infections in co-culture and mouse model
Source: Front Bioeng Biotechnol. 2023 Oct 19;11:1227148. doi: 10.3389/fbioe.2023.1227148 (PMC10621036; doi:10.3389/fbioe.2023.1227148)
Supplement: Supplementary file 1 [file DataSheet1.docx]

Supplementary Material

Effect of titanium implants along with silver ions and tetracycline on type I interferon-beta expression during implant-related infections in co-culture and mouse model

Muhammad Imran Rahim^1*^, Syed Fakhar-ul-Hassnain Waqas^2^, Stefan Lienenklaus^3^, Elmar Willbold^4^, Michael Eisenburger^1^, Meike Stiesch^1^

*** Correspondence:**

Muhammad Imran Rahim
[Rahim.Muhammad@mh-hannover.de](mailto:Rahim.Muhammad@mh-hannover.de)

**
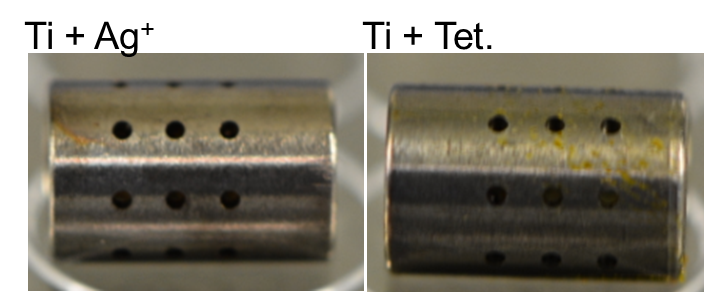
**

**Supplementary Figure 1.** Surface morphology of cylindrical titanium implants coated with silver ions (Ti+Ag^+^) and tetracycline (Ti+Tet.).


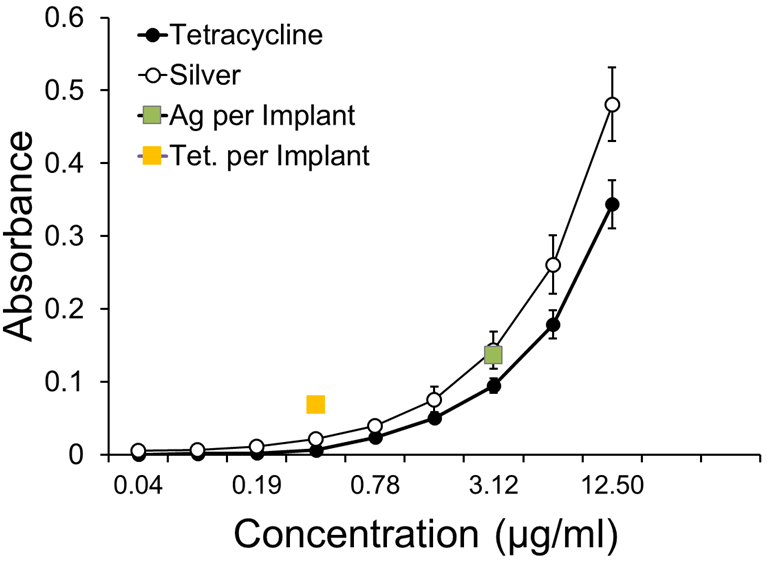


**Supplementary Figure 2.** Absorbance values showing standard curve of silver nitrate (white squares) and tetracycline (black squares) at various concentrations. Green box shows the absorbance of silver ions (Ag^+^) collected as supernatant from silver ions-coated implants after 24 hours of incubation. Orange box indicates the absorbance of supernatants collected from tetracycline-coated implants after 24 hours of incubation.

**
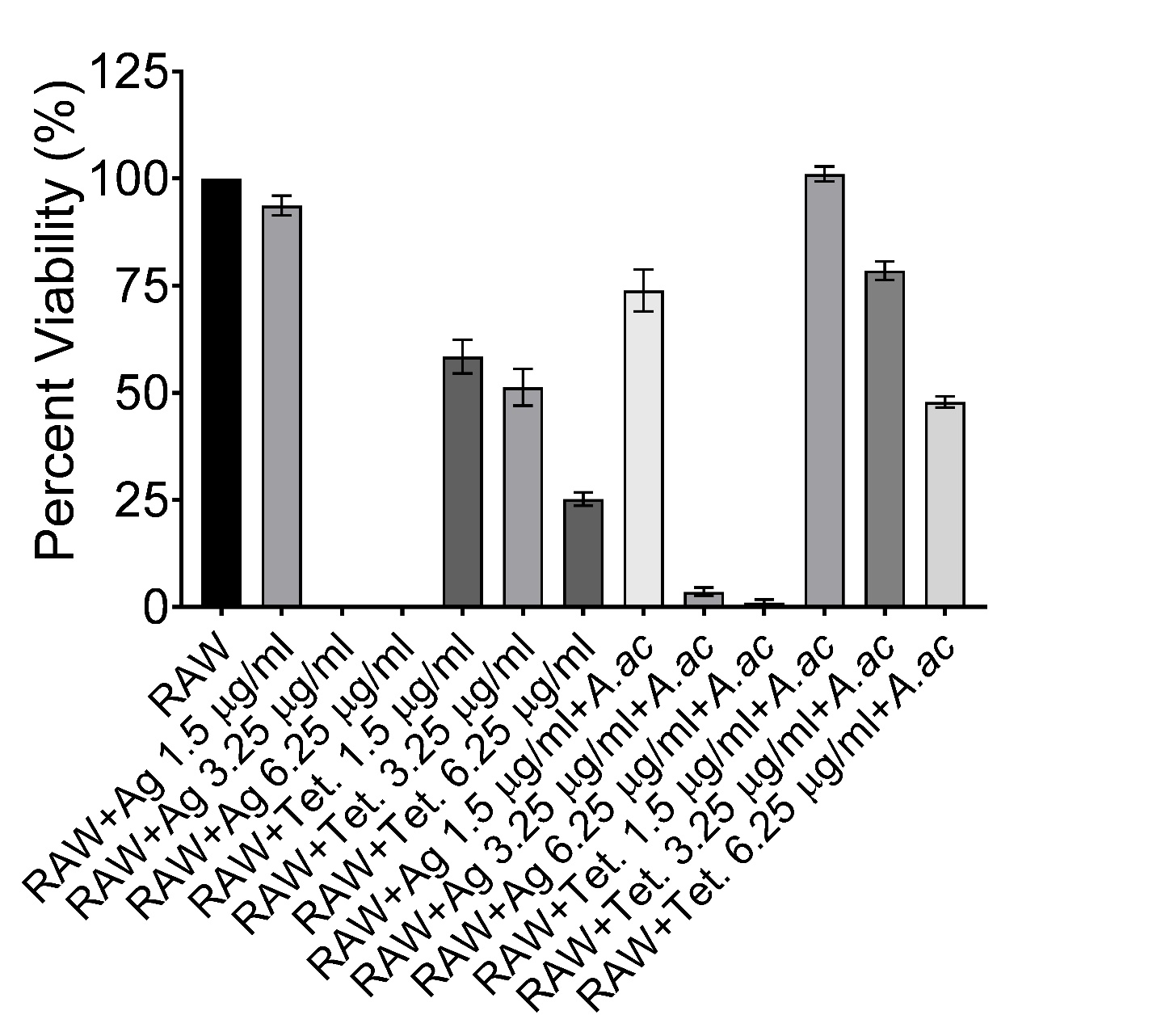
**

**Supplementary Figure 3.** Quantitative cytotoxicity of macrophages. Macrophages were incubated with 1.5 μg/ml, 3.25 μg/ml and 6.25 μg/ml of silver nitrate and tetracycline. After 48 hours of incubation, toxicity of cells was measured.


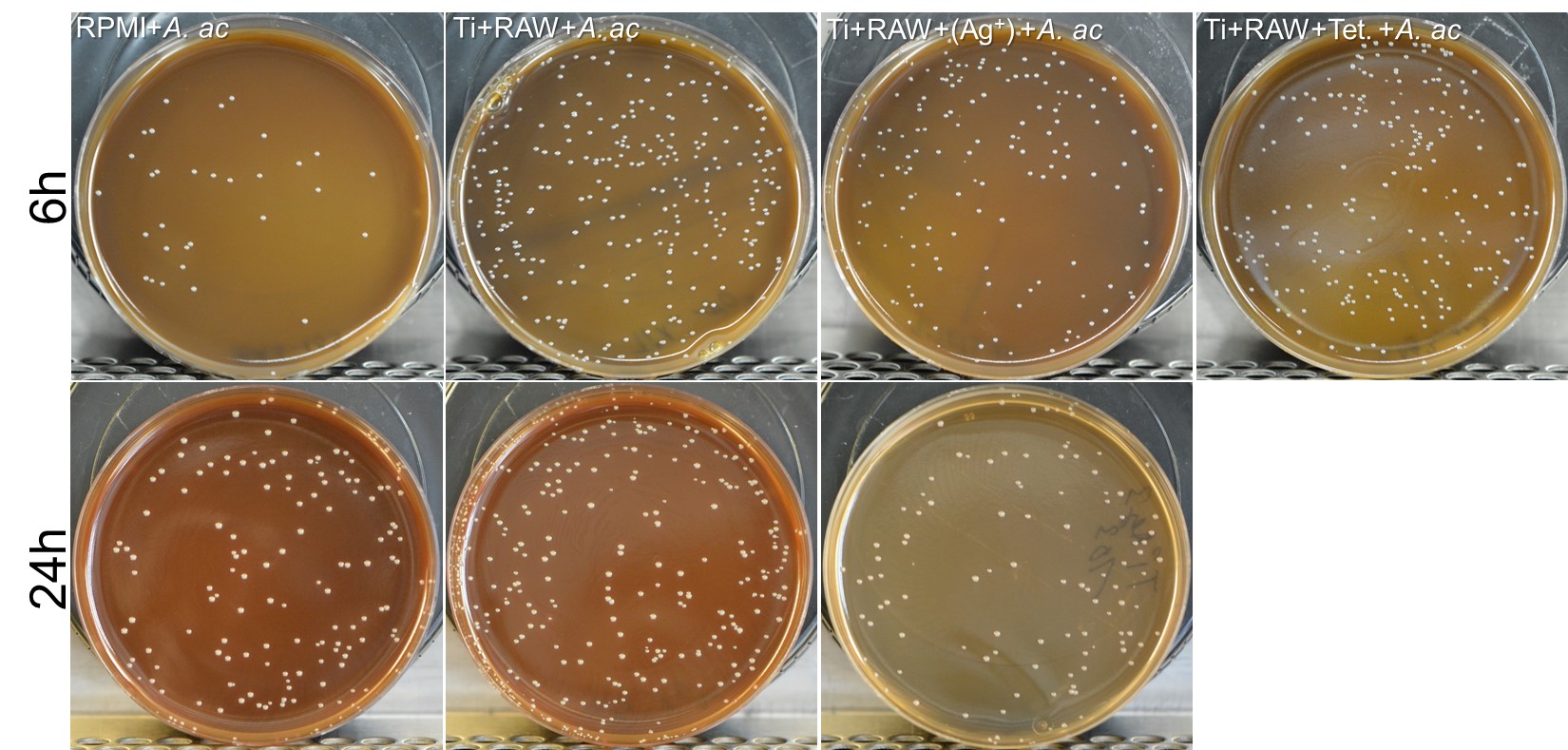


Supplementary Figure 4. The growth of A. actinomycetemcomitans on FAA plates supplemented with 5% sheep blood from the cell culture medium (RPMI+A. ac), medium with macrophages on titanium (Ti+RAW+A. ac), medium with macrophages on Ag^+^-coated titanium (Ti+RAW+(Ag^+^)+A. ac) and medium with macrophages on tetracycline-coated titanium (Ti+RAW+Tet.+A. ac) after 6h and 24h of incubations.


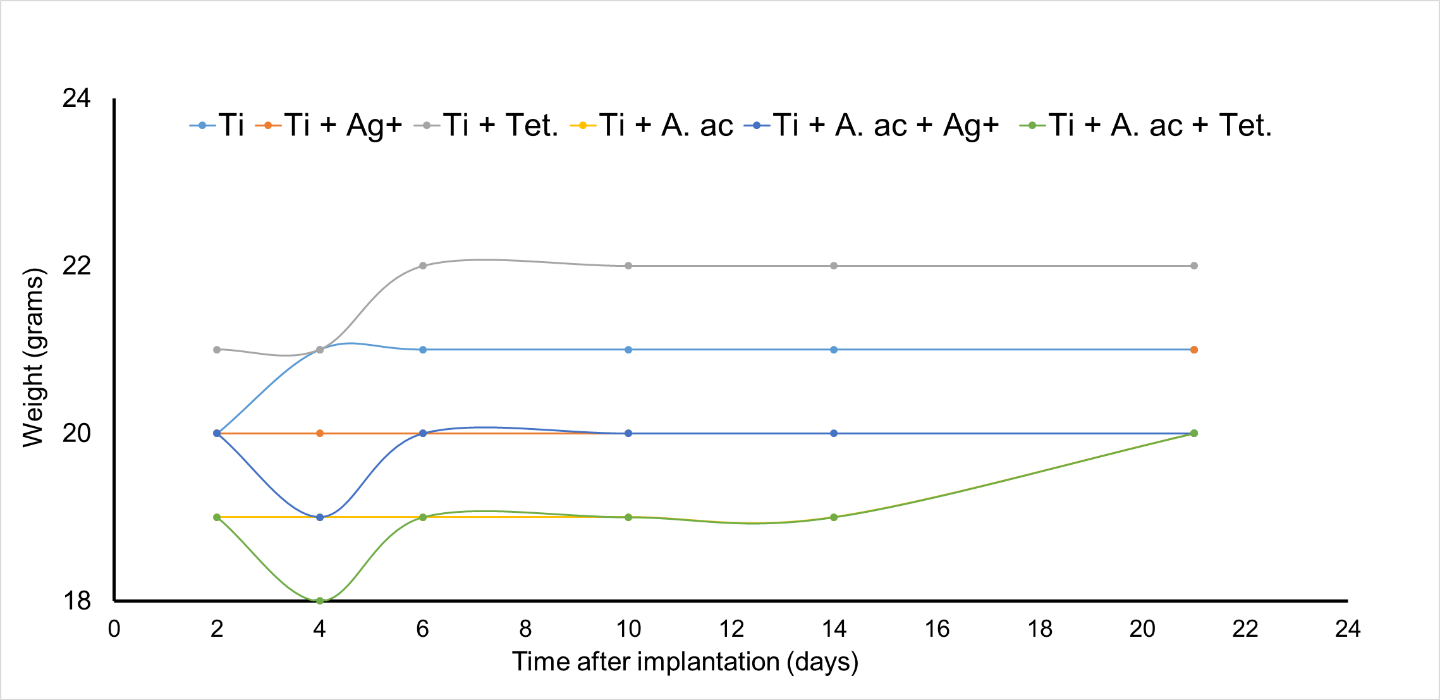


**Supplementary Figure 5.** Changes in the weight of animals bearing sterile titanium (Ti), silver ions-coated titanium (Ti + Ag^+^), tetracycline-coated titanium (Ti + Tet.), *A. actinomycetemcomitans* infected titanium (Ti + *A. ac*), silver ions-coated titanium (Ti + *A. ac* + Ag^+^), and tetracycline-coated titanium (Ti + *A. ac* + Tet.) implants at the indicated time points.


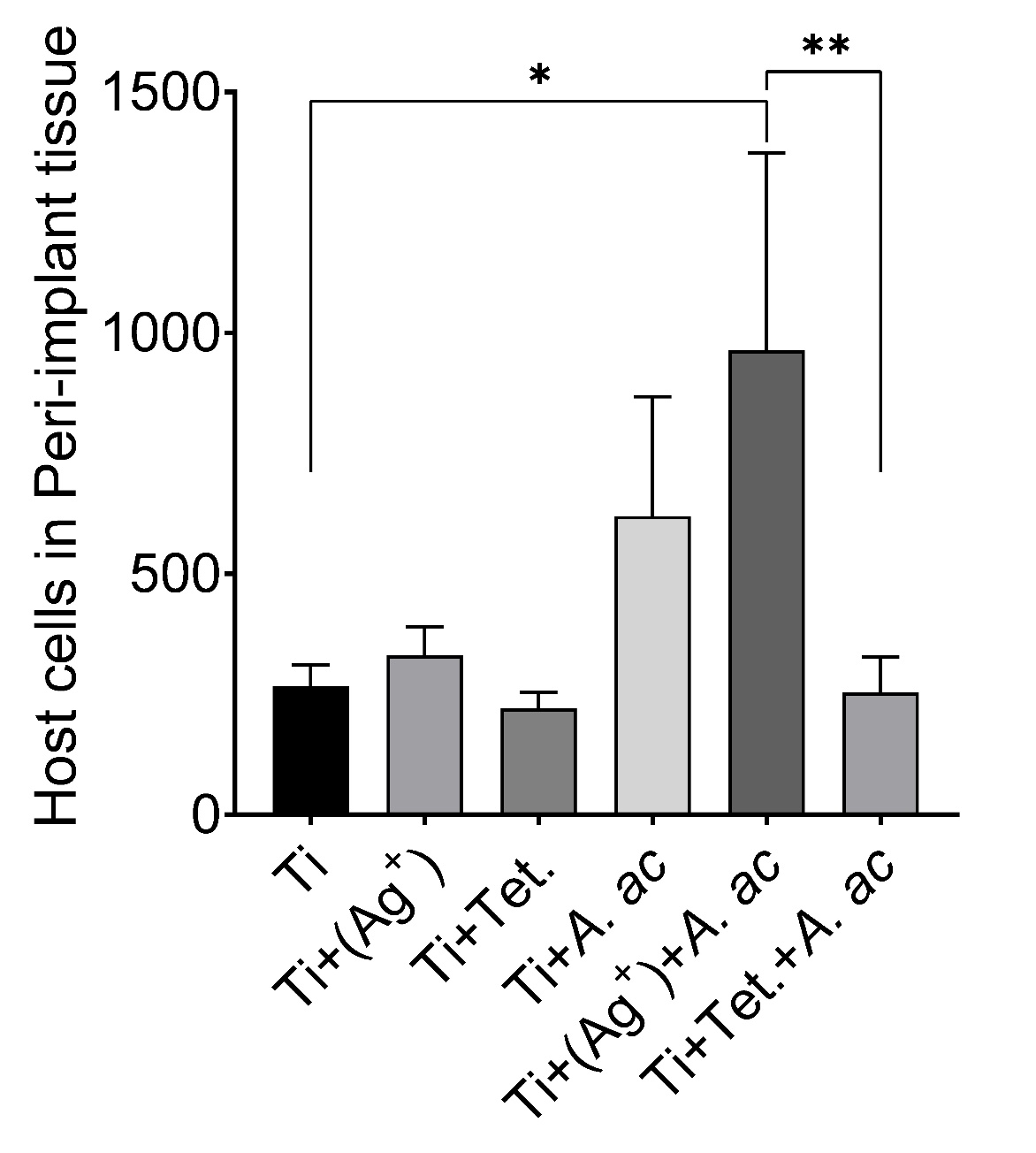


**Supplementary Figure 6.** Number of host cells in the peri-implant tissue interfaces adjacent to titanium (Ti), Ag^+^-coated titanium (Ti+(Ag^+^), tetracycline-coated titanium (Ti+Tet.), and titanium infected with *A. actinomycetemcomitans* (Ti+*A. ac*), *A. actinomycetemcomitans* infected Ag^+^-coated titanium (Ti+(Ag^+^)+*A. ac),* and *A. actinomycetemcomitans* infected tetracycline-coated titanium (Ti+Tet.+*A. ac*). The cells were counted from three different histology images using image J software. Bars depict mean ± SD. Statistical analysis was performed with a Graph pad prism by using the One-Way ANOVA test.

**
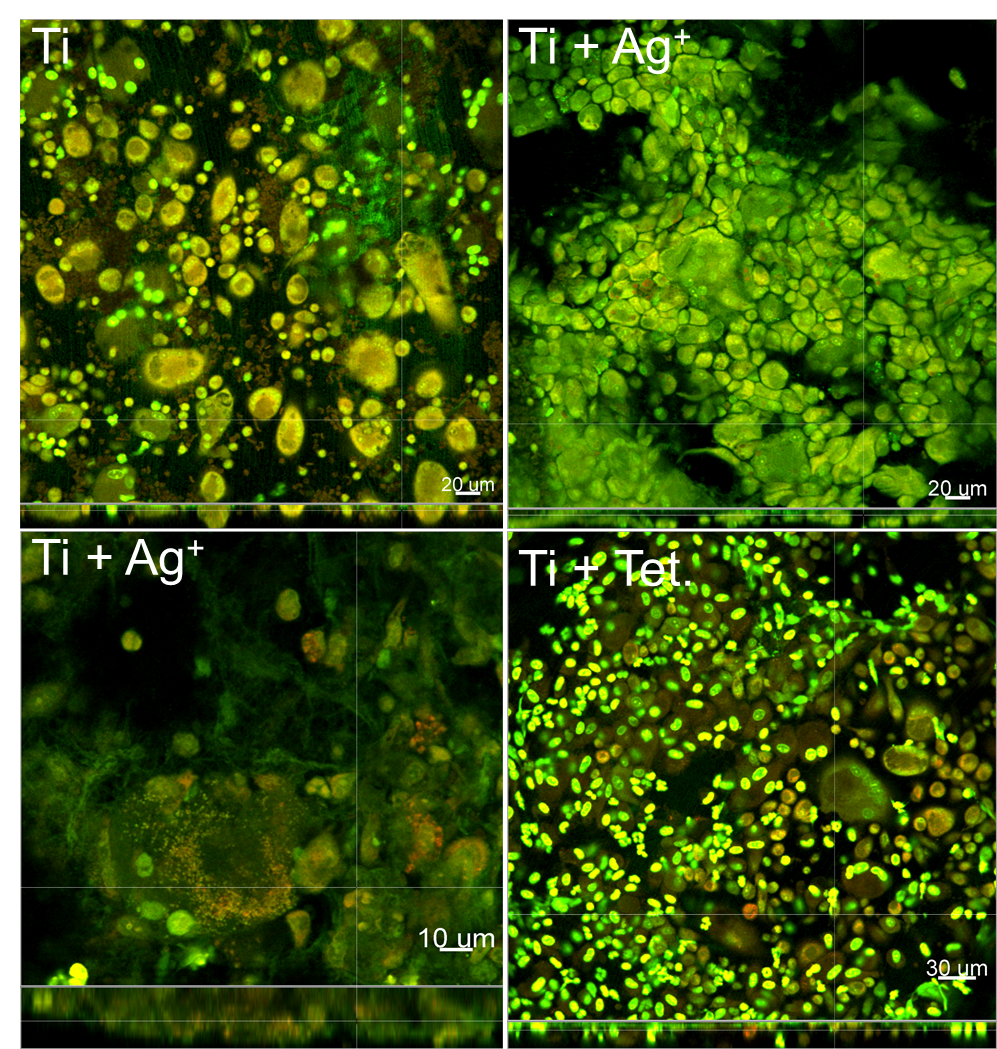
**

**Supplementary Figure 7.** Surface morphologies of *A. actinomycetemcomitans* infected cylindrical titanium (Ti), Ag^+^-coated titanium (Ti + Ag^+^) and tetracycline-coated titanium (Ti + Tet.) implants after three weeks of implantation.

**Table S1 Sequence of primers used for quantitative real-time PCR (RT-qPCR)**

| Primers | Sequence (5 to 3) |
| --- | --- |
| β-actin Forward | GGCTGTATTCCCCTCCATCG |
| β-actin Reverse | CCAGTTGGTAACAATGCCATGT |
| IFN-β Forward | CTGGCTTCCATCATGAACAA |
| IFN-β Reverse | CATTTCCGAATGTTCGTCCT |
| IFN-α Forward | TACTCAGCAGACCTTGAACCT |
| IFN-α Reverse | CAGTCTTGGCAGCAAGTTGAC |
| ISG15 Forward | GAGCTAGAGCCTGCAGCAAT |
| ISG15 Reverse | TTCTGGGCAATCTGCTTCTT |
| GAPDH Forward | GTGGCAAAGTGGAGATTGTT |
| GAPDH Reverse | CTTGACTGTGCCGTTGAATT |
| MX2 Forward | TCACCAGAGTGCAAGTGAGG |
| MX2 Reverse | CATTCTCCCTCTGCCACATT |
| MX1 Forward | TGGACATTGCTACCACAGAGGC |
| MX1 Reverse | TTGCCTTCAGCACCTCTGTCCA |
| IL-6 Forward | TACCACTTCACAAGTCGGAGGC |
| IL-6 Reverse | CTGCAAGTGCATCATCGTTGTTC |
| TLR2 Forward | GCAAACGCTGTTCTGCTCAG |
| TLR2 Reverse | AGGCGTCTCCCTCTATTGTATT |
| TLR4 Forward | ATGCATGGATCAGAAACTCAGCAA |
| TLR4 Reverse | AAACTTCCTGGGGAAAAACTCTGG |
| IRF3 Forward | TTGTGATGGTCAAGGTTGTTCC |
| IRF3 Reverse | TGGAGGTAGGCCTTGTACTGGT |
| IRF7 Forward | ACCGTGTTTACGAGGAACCC |
| IRF7 Reverse | ACCGTGTTTACGAGGAACCC |
| CXCL1 Forward | TCCAGAGCTTGAAGGTGTTGCC |
| CXCL1 Reverse | AACCAAGGGAGCTTCAGGGTCA |
| TNF-α Forward | GCCCACGTCGTAGCAAACCACCAA |
| TNF-α Reverse | ACACCCATTCCCTTCACAGAGCAAT |
